# Characterization of two types of cesium-bearing microparticles emitted from the Fukushima accident via multiple synchrotron radiation analyses

**DOI:** 10.1038/s41598-020-68318-2

**Published:** 2020-07-21

**Authors:** Hikaru Miura, Yuichi Kurihara, Masayoshi Yamamoto, Aya Sakaguchi, Noriko Yamaguchi, Oki Sekizawa, Kiyofumi Nitta, Shogo Higaki, Daisuke Tsumune, Takaaki Itai, Yoshio Takahashi

**Affiliations:** 1 Atmospheric and Marine Environmental Sector, Environmental Science Research Laboratory, Central Research Institute of Electric Power Industry, 1646 Abiko, Abiko-shi, Chiba 270-1194 Japan; 2 Ningyo-Toge Environmental Engineering Center, Japan Atomic Energy Agency, 1550 Kamisaibara, Kagamino-cho, Tomata-gun, Okayama 708-0698 Japan; 3 Low Level Radioactivity Laboratory, Kanazawa University, Kanazawa, Ishikawa 923-1224 Japan; 4 Center for Research in Isotopes and Environmental Dynamics, University of Tsukuba, 1-1-1, Tennodai, Tsukuba, Ibaraki 305-8577 Japan; 5 Institute for Agro-Environmental Sciences, NARO, 3-1-3, Kannondai, Tsukuba, Ibaraki 305-8604 Japan; 6 Japan Synchrotron Radiation Research Institute (JASRI), 1-1-1 Kouto, Sayo-cho, Sayo-gun, Hyogo 679-5198 Japan; 7 Isotope Science Center, The University of Tokyo, 2-11-16 Yayoi, Bunkyo-ku, Tokyo, 113-0032 Japan; 8 Department of Earth and Planetary Science, Graduate School of Science, The University of Tokyo, 7-3-1 Hongo, Bunkyo-ku, Tokyo, 113-0033 Japan

**Keywords:** Environmental sciences, Environmental chemistry

## Abstract

A part of radiocesium emitted during the Fukushima nuclear accident was incorporated in glassy water-resistant microparticles, called Type-A particles, which are spherical with ~ 0.1 to 10 µm diameter and ~ 10^–2^ to 10^2^ Bq cesium-137 (^137^Cs) radioactivity; they were emitted from Unit 2 or 3 of the Fukushima Daiichi Nuclear Power Plant. Meanwhile, Type-B particles, having various shapes, 50–400 µm diameter, and 10^1^–10^4^ Bq ^137^Cs radioactivity, were emitted from Unit 1. The chemical properties of these radioactive particles have been reported in detail, but previous studies investigated only a small number of particles, especially Type-B particles. We tried to understand radioactive particles systematically by analyzing a large number of particles. Micro-X-ray computed tomography combined with X-ray fluorescence analysis revealed the presence of many voids and iron-rich part within Type-B particles. The ^137^Cs concentration (Bq mm^–3^) of Type-A particles was ~ 10,000 times higher than that of Type-B particles. Among the Type-B particles, the spherical ones had higher concentration of volatile elements than the non-spherical ones. These differences suggested that Type-A particles were formed through gas condensation, whereas Type-B particles were formed through melt solidification. These findings might contribute to the safe decommissioning of reactors and environmental impact assessment.

## Introduction

On March 11, 2011, a great earthquake hit the eastern part of mainland Japan and triggered several gigantic tsunami waves, attacking the six-unit Fukushima Daiichi Nuclear Power Plant (FDNPP). The tsunamis damaged the electric functions to cool reactor cores and rapidly increased the temperature in the primary containment vessels (PCVs) in Units 1–3 that were operating during the accident. In addition, the chemical reactions of water and zirconium for fuel cladding at high temperature increased the pressure inside the reactors due to the large amount of hydrogen (H) gas produced. The Tokyo Electric Power Company (TEPCO) tried to vent H and other gases to decrease the pressure. According to TEPCO^1^, H explosions occurred in Units 1 (15:36 JST, March 12, 2011) and 3 (11:01 JST, March 14, 2011). By contrast, the pressure of PCV in Unit 2 decreased, possibly due to a break, thus causing gas emission from the blowout panel of the reactor building on the morning of March 15, 2011. As a result, large amounts of radionuclides in the nuclear reactors of FDNPP were released into the environment. Nine years after the accident, the internal conditions of these units remain unknown.

Among the emitted radionuclides, cesium-137 (^137^Cs, *T*_1/2_ = 30.2 y) has been widely studied because of its long half-life and its large amount emitted to the environment. Among the ^137^Cs emitted to the atmosphere, 12–15 PBq of ^137^Cs was deposited on the Pacific, whereas 3–6 PBq accumulated on the land through dry and wet depositions^2^. Kaneyasu et al.^3^ suggested sulfate aerosol as a potential transport medium for soluble Cs. Adachi et al.^4^ found water-resistant glassy particles containing Cs, radiocesium-bearing microparticles (CsMPs; this type of CsMPs can be called Type-A particles, as suggested by Igarashi et al.^5^), from an aerosol filter in Tsukuba City, Japan collected during 21:10 JST (March 14, 2011) to 09:10 JST (March 15, 2011). Subsequent studies^6–13^ have revealed that the Type-A particles are spherical with ~ 0.1 to 10 μm size and ~ 10^–2^ to 10^2^ Bq of ^137^Cs. Ono et al.^14^ first reported a new type of CsMPs (Type-B particles in the paper of Igarashi et al.^5^) collected at the vicinity of FDNPP. Subsequently, Satou et al.^15^ investigated Type-B particles. These studies showed that Type-B particles are similar to Type-A particles because their main chemical component is silicon dioxide (SiO_2_), but heterogeneous distributions of various elements were suggested. Moreover, Type-B particles have various shapes with porous surface features, and their ^137^Cs activity is considerably higher than that of Type-A particles due to their larger size. Table 1 shows a summary of Type-A and -B particles^5,13^. A recent review has summarized that Type-A and -B particles are the main forms of radiocesium emitted as CsMPs from FDNPP^5^. However, further characterization of CsMPs is needed because the classification of CsMPs is essential to describe the FDNPP accident in terms of radiocesium emission. This information should be passed onto the next generation for possible similar nuclear incidents.

**Table 1:** Summary of Type-A and -B particles^5,13^.

	Type-A particle	Type-B particle
Origin expected from ^134^Cs/^137^Cs activity ratio	Unit 2/3	Unit 1
Size	~ 0.1 to 10 µm	~ 50 to 400 µm
Shape	Spherical	Various shapes
^137^Cs radioactivity	~ 10^–2^–10^2^ Bq/particle	~ 10^1^–10^4^ Bq/particle

Most studies have dealt with a small number of CsMPs, typically less than five particles, thus inhibiting the elucidation of common conclusions from their analytical results. To solve this problem, the current work introduced the following two approaches. (i) We have established a quick separation method for CsMPs, wet separation method; this method is based on the fact that CsMPs are water-resistant within a short time^16,17^. (ii) The wet separation method was applied to road dust samples with high contents of radiocesium that were collected before decontamination activities in the area within 45 km from FDNPP, as reported by Yamamoto et al.^18^ As a result, we successfully analyzed 10 Type-A and 57 Type-B particles, thus allowing us to establish general conclusions on the physicochemical characteristics of CsMPs.

Another strategy is to conduct synchrotron radiation-based µ-X-ray computed tomography (µ-X-ray CT) and X-ray fluorescence (XRF) analyses by using X-ray microbeam. Martin et al.^19^ showed internal structure and elemental distribution via µ-X-ray CT. Micro-X-ray CT was also effectively used in the current study because the most decisive difference between Type-A and -B particles is their morphologic features, namely, their nonporous and porous natures, respectively. Consequently, Type-A particles were successfully distinguished from Type-B particles by using their chemical and morphological characteristics, thus allowing us to understand the conditions of each unit at the accident when CsMPs were formed. This information contributes to the understanding of radiocesium-bearing materials inside reactors and the decommissioning of the reactors in the future.

## Results and discussion

### Sample characterization (Type-A and -B particles)

**Figure 1:**
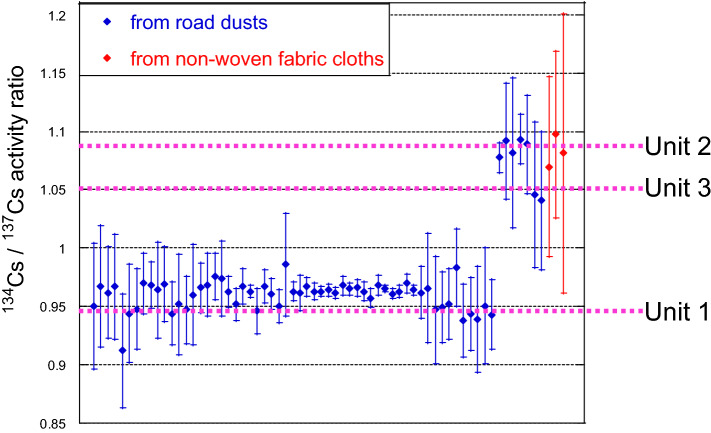
^134^Cs/^137^Cs activity ratio of Type-A and -B particles separated from road dusts and non-woven fabric cloths. The values of the damaged reactor cores (Units 1–3) are cited from Nishihara et al.^20^.

**Figure 2:**
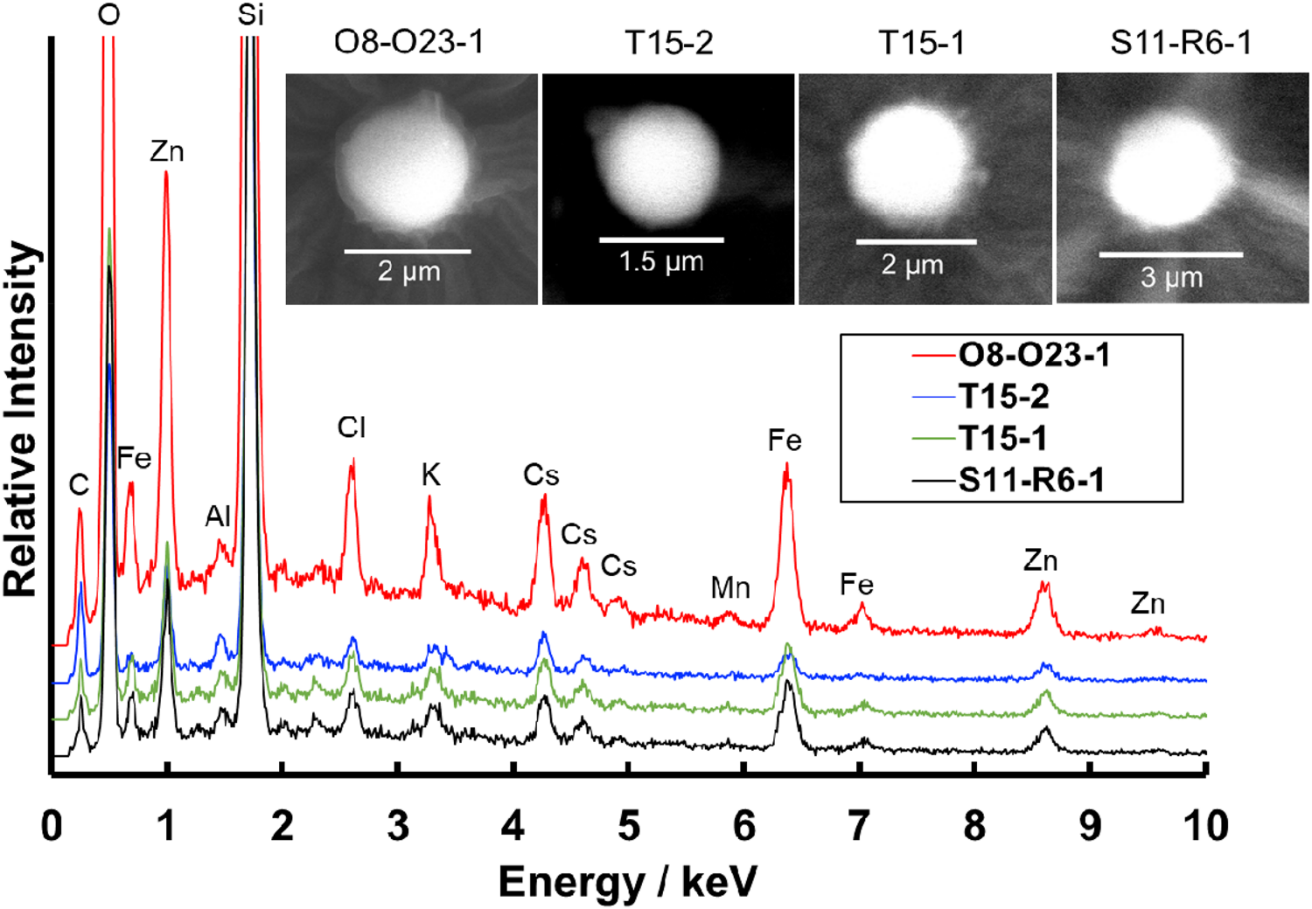
Examples of the SEM and EDS results of Type-A particles. Carbon is from carbon coating.

The ^134^Cs/^137^Cs activity ratio of each separated CsMP measured through gamma-ray spectrometry with a high-purity germanium semiconductor detector (HPGe) is shown in Fig. 1. The calculated Cs activity ratios were ~ 0.94 for Unit 1, ~ 1.08 for Unit 2, and ~ 1.05 for Unit 3^20^. On the basis of these ratios, we could determine the unit from which each CsMP originated. CsMPs from (i) Unit 1 were Type-B particles, and those from (ii) Unit 2 or 3 (written as Unit 2/3 hereinafter) were Type-A particles. Type-A and -B were also related to their size, morphology, and chemical compositions^5,15^. The results of scanning electron microscopy (SEM) with energy-dispersive spectrometer (EDS) measurement for Type-A particles showed that their shape was almost spherical, and their chemical composition included oxygen (O), silicon (Si), Cs, iron (Fe), zinc (Zn), chlorine (Cl), and potassium (K, Fig. 2). This finding is similar to those reported for other Type-A particles^4,7^. The ^137^Cs activities of Type-A particles ranged from 0.67 to 62 Bq.

**Figure 3:**
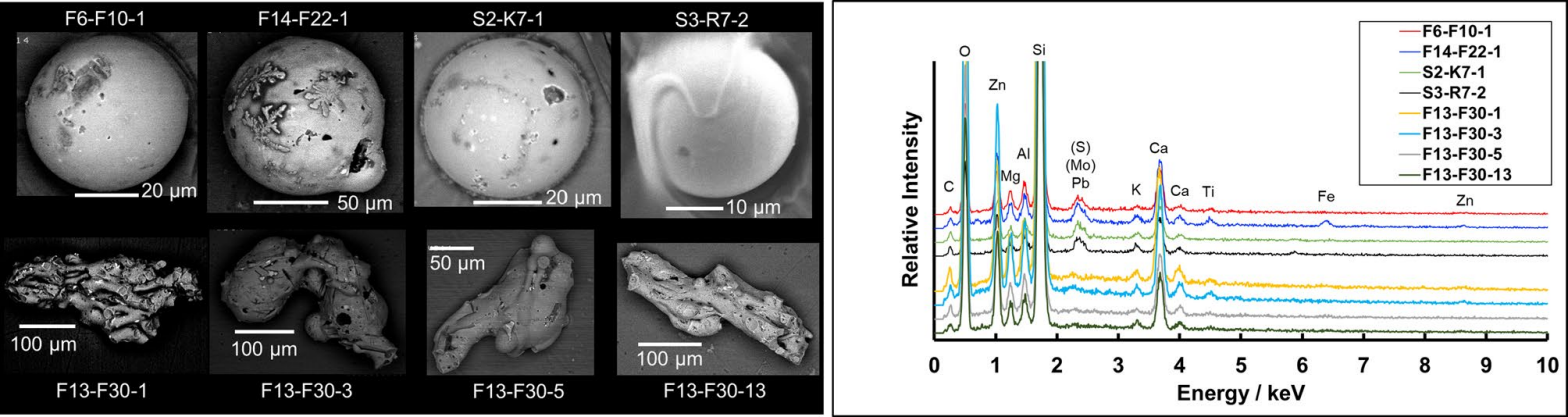
Examples of the SEM and EDS results of Type-B particles. The upper SEM figures are spherical Type-B particles, and the lower figures are non-spherical Type-B particles. Carbon is from carbon coating.

Figure 3 shows that Type-B particles had various shapes. We found 18 spherical (including approximately spherical) and 39 non-spherical Type-B particles. The analysis of a number of particles helped us classify Type-B particles in accordance with their shapes. The surface of non-spherical Type-B particles had many holes, possibly due to degassing. The EDS result indicated that the chemical composition of Type-B particles (Fig. 3) included O, Si, magnesium, aluminum (Al), Fe, Zn, and calcium (Ca), which are similar to those reported for other Type-B particles^14,15^. The ^137^Cs activities of Type-B particles ranged from 3.23 Bq to 1.32 × 10^4^ Bq, but Cs was not detected via EDS due to its low concentration. In addition, antimony-125 (^125^Sb) was quantified in Type-B particles through gamma spectrometry with a HPGe with radioactivities ranging from 0.8 to 26.4 Bq.

The differences between (i) Type-A and -B particles and (ii) spherical and non-spherical Type-B particles are discussed in the following sections.

### Inner structure and volume of Type-B particles by µ-X-ray CT analysis

**Figure 4:**
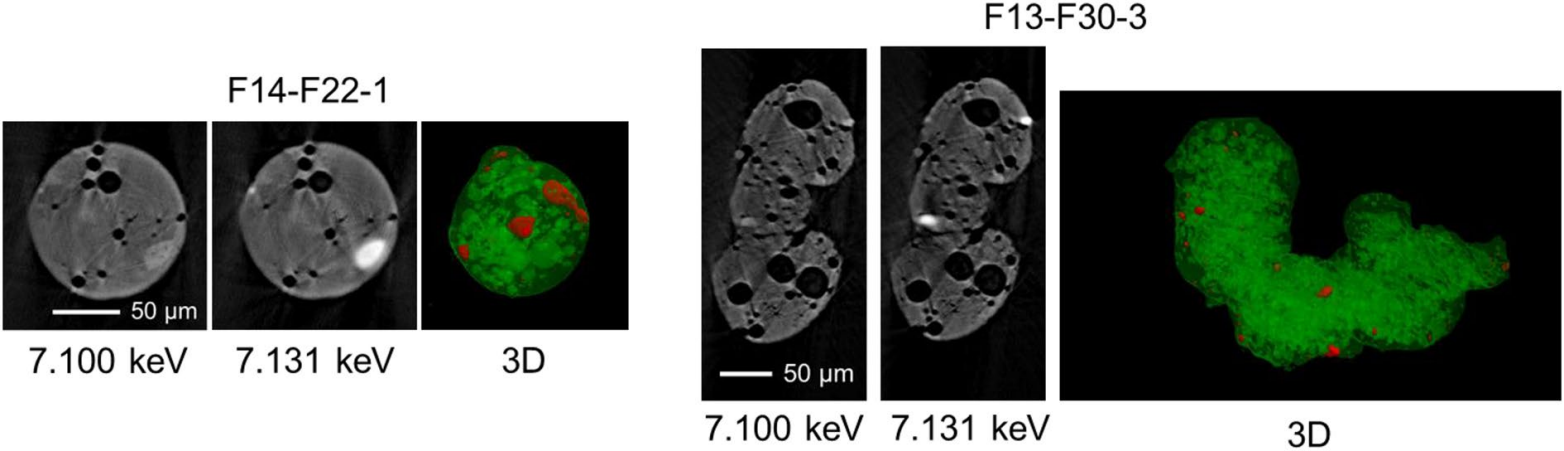
Slice of a µ-X-ray CT image taken at incident X-ray energy below and above Fe K-edge emission for spherical (left) and non-spherical (right) Type-B particles. 3D images are constructed using ImageSurfer (https://www.imagesurfer.org). Many voids exist. The white areas in slice images and the red areas in 3D images indicate Fe-rich parts.

Figure 4 shows the slices from the µ-X-ray CT results for Type-B particles with a number of voids in their structures as found for volcanic pumice^21^. The white areas with CT image absorbed X-ray to a larger degree than the matrix area did, thus reflecting the presence of heavy elements in Type-B particles. Micro-X-ray CT measurement was conducted below and above Fe and Zn K-edge emissions. In Fig. 4, most of the white areas disappeared at 7.1 keV (below Fe K-edge), indicating that they contained Fe. This observation is also evident in the 3D maps constructed from the slices (Figs. 4 and Supplementary Fig. S1). Fe was observed by backscattered electron imaging of SEM. Chromium and nickel with Fe were detected using EDS spectra (Supplementary Fig. S2) and considered fragments of stainless steel used in the units^14^. Kogure et al.^7^ showed that Fe is homogenously distributed in Type-A particles. In addition, Furuki et al.^9^ showed that Zn–Fe oxide nanoparticles are present in Type-A particles. By contrast, the current results revealed that Fe was heterogeneously distributed as fragments in Type-B particles. The absorption intensity of the white areas including Fe did not change below and above the Zn K-edge, indicating that Fe with a heterogeneous distribution might not be present as Zn–Fe oxide for Type-B particles.

**Figure 5:**
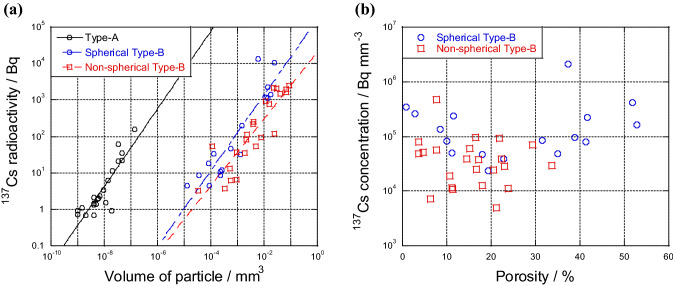
Relationships between (**a**) the ^137^Cs radioactivity and volume of CsMPs calculated through µ-X-ray CT and (**b**) the ^137^Cs concentration and porosity of Type-A and -B particles. The blue circles and red squares indicate spherical and non-spherical Type-B particles, respectively. Part of the data for Type-A particles are referred to Adachi et al.^4^, Abe et al.^6^, Satou et al.^22^, and Furuki et al.^9^.

Determining the volume of Type-B particles through external shape is difficult because voids cannot be externally observed via SEM. Micro-X-ray CT images enabled us to calculate the volume accurately. In this calculation, the µ-X-ray CT images measured at 9.8 keV were used, and the voids in the particles were excluded from the volume, because images at high energy are clear due to the low X-ray absorption. The volume of Type-A particles, which were assumed to be spherical, was calculated using apparent diameter. Figure 5 shows the relationship between the ^137^Cs radioactivity and volume of Type-A and -B particles, including some data of Type-A reported by Adachi et al.^4^, Abe et al.^6^, Satou et al.^22^, and Furuki et al.^9^. The blue circles and red squares represent the data for spherical and non-spherical Type-B particles, respectively. Large particles generally had high ^137^Cs radioactivity for Type-A and -B particles. The ^137^Cs concentration (Bq mm^–3^) of Type-A particles was ~ 10,000 times higher than that of Type-B particles. Kogure et al.^7^ showed that Type-A particles include 7.2–11.7 (wt%) Cs_2_O by analyzing three samples, suggesting that Cs_2_O in Type-B particles is approximately 10 ppm. Voids and ^137^Cs concentration are important to characterize the differences between Type-A and -B particles. In addition, a difference in ^137^Cs concentration between spherical and non-spherical Type-B particles was found. Spherical Type-B particles had ~ 10 times higher ^137^Cs concentration than non-spherical Type-B particles. The gradients of approximately straight lines of Type-A and -B particles on the logarithmic graph were almost 1. This result might suggest that ^137^Cs was distributed homogeneously in CsMPs because the ^137^Cs radioactivity of CsMPs was proportional to their volume. However, Kogure et al.^7^ showed that alkali ions, including Cs, in Type-A particles are radially distributed, although the degree of radial dependence is considerably different among varying particles.

We calculated the porosity of Type-B particles by using µ-X-ray CT images. Figure 5 shows the relationship between porosity and ^137^Cs concentration. The variation range of porosity for non-spherical Type-B particles was from 3.7% to 34%, whereas that for spherical Type-B particles was from 0.78% to 53%. In particular, spherical Type-B particles with porosity larger than 30% had high ^137^Cs concentration.

### Difference between spherical and non-spherical Type-B particles from the viewpoint of the volatility of elements

Thirteen spherical and 21 non-spherical Type-B particles were measured through μ-XRF analysis at BL-4A of the Photon Factory, KEK. Various elements were analyzed, but the results of light elements that emitted XRF with low energy were not good. In some elements such as Cl, K, Ca, and manganese (Mn), higher concentration areas were observed at the right side than at the left side of Type-B particles (Supplementary Fig. S3). This observation was caused by the position of the detector, which was located at the right side in this beamline. XRF with low energy from the light elements located at the left side of Type-B particles could not reach the detector due to self-absorption by Type-B particles; we did not prepare thin sections to have a flat surface for the measurement. Other elements, such as Fe, had an extremely high-concentration area, as suggested by µ-X-ray CT. By contrast, the results of rubidium (Rb, Kα_1_: 13.4 keV) and strontium (Sr, Kα_1_: 14.1 keV) were good due to the XRF of the elements with high XRF energy that were not subjected to the self-absorption effect. Consequently, these elements exhibited an almost homogenous distribution compared with light elements. We assumed that the matrix of Type-B particles was SiO_2_. The attenuation length (the depth where the intensity of X-rays falls to 1/e of its original intensity) was longer than 500 μm when the X-ray energy was above 13 keV. Therefore, Rb and Sr with high energy XRF were selected for analysis.

**Figure 6:**
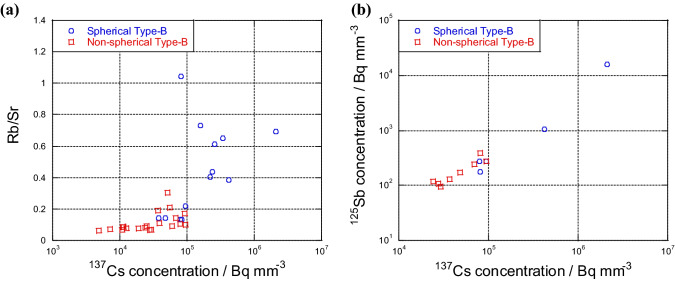
(**a**) Relationship between the Rb/Sr intensity ratio of XRF Kα peaks and ^137^Cs concentration. (**b**) Relationship between ^125^Sb and ^137^Cs concentrations. The blue circles and red squares indicate the spherical and non-spherical Type-B particles, respectively.

Allègre et al.^23^ showed the order of volatility of various elements in terms of cosmochemical classification of elements. Pontillon et al.^24^ also indicated the volatility of fission products. Rb, Sb, and Cs served as volatile elements, and Sr served as a refractory element. We determined the Rb/Sr ratio from the average XRF count of Rb and Sr to discuss the relationship of Rb/Sr with ^137^Cs and ^125^Sb concentrations (Fig. 6).

From the volatility of these elements, spherical Type-B particles had higher Rb/Sr ratio than non-spherical Type-B particles. The Rb/Sr ratio in the spherical Type-B particles increased with ^137^Cs concentration. Therefore, spherical Type-B particles possibly had a higher concentration of volatile elements, including Cs, compared with non-spherical Type-B particles. The relationship between ^137^Cs and ^125^Sb concentrations (Fig. 6) revealed that (i) Type-B particles with a high ^137^Cs concentration had a high ^125^Sb concentration, and (ii) the Sb concentration of spherical Type-B particles was higher than that of non-spherical Type-B particles.

A similar phenomenon was observed in the particles from the nuclear test. Bonamici et al.^25^ analyzed the particles made by Trinity that are similar to Type-B particles in terms of size, shape, and elemental composition. They showed a relationship between the volatility index {= (Na + K)/(Al + Ca + Ti)} and concentrations of some elements. With increasing volatility index of particles, the concentrations of volatile elements including Rb and Cs also increased, whereas those of refractory elements including Sr decreased. Particles from the nuclear test were formed at high temperature and pressure, causing the fractionation of volatile and refractory elements, such as CsMPs. The Rb/Sr ratio could be used as a signature similar to the volatility index because Rb and Sr have similar volatile–refractory characteristics to (Na + K) and Ca, respectively.

### Implications on the generation process

The difference in ^137^Cs activity per volume between Type-A and -B particles indicated that they were generated through gas condensation and melt solidification, respectively. On the basis of ^134^Cs/^137^Cs activity ratio, Type-A particles were from Unit 2/3. IRID^26^ concluded that Type-A particles were from Unit 2 of FDNPP because Zn, K, and a part of Si in Type-A particles originated from the inorganic coating of the wall in the suppression chamber (S/C). Cooling water from S/C for fuel rod was used by Unit 2. In Unit 2, gas emission from the blowout panel (morning of March 15) was reported by TEPCO^1^, by which large amounts of radionuclides were emitted. Relatively volatile elements, such as Cs, K, Cl, and Si, in the unit were evaporated under high-temperature condition and emitted through the blowout panel. Type-A particles were generated through gas condensation from SiO vapor^26^, causing their spherical form.

From ^134^Cs/^137^Cs activity ratio, Type-B particles were emitted from Unit 1, where H explosion occurred^1^. Fuel in Unit 1 experienced melt-through, and molten core–concrete interaction possibly occurred^26^. The heat insulation material in the wall of the operation floor in Unit 1 was possibly melted due to the high-temperature and -pressure conditions^26,27^. Before explosion, these molten materials and gas of volatile elements, such as H, Cs, Sb, and Rb, were possibly present within Unit 1. A part of the gas was probably dissolved into the molten materials, as suggested by Cs incorporation onto the surface of Type-A particles in the report of Kogure et al.^7^. Finally, the explosion caused the emission of gas and molten materials, including dissolved volatile elements, to the surrounding environment. We speculated that a part of dissolved volatile elements became gaseous species in the melt due to a decrease in pressure. A part of the gaseous species remained within particles to form voids within them because the surface was solidified faster by rapid cooling than the interior. Particles with high porosity had high ^137^Cs radioactivity by capturing large amounts of volatile elements, including Cs.

Among Type-B particles, spherical and non-spherical particles had clearly different chemical compositions; volatile (Rb, Sb, and Cs) and refractory (Sr) elements were more abundant in spherical and non-spherical particles, respectively. The higher density of holes on the surface of non-spherical particles than that on spherical particles suggested the difference in the cooling rate of the two particle types. The non-spherical particles, possibly formed on the surface of structural materials at high temperature in the reactor, cooled at a low rate, which resulted in the release of gaseous species from the surface at high temperature. On the contrary, voids were found in the interior of the spherical particles with a fast cooling rate, which should be formed in the free space in the reactor. Thus, the release of gaseous species, including Cs, to form surface holes caused lower concentrations of ^137^Cs and ^125^Sb and a smaller Rb/Sr ratio for the non-spherical than those for spherical particles. Some of the non-spherical Type-B particles did not seem to melt completely because we could observe fibrous materials possibly from the heat insulation material made of highly fibrous Si-rich materials (Fig. 5), as reported by Martin et al.^27^ and Satou et al.^15^. This result suggested that the non-spherical particles were formed on the surface of the structural materials in the reactor. Thus, gas of volatile elements might not dissolve to a large degree into non-spherical Type-B particles due to incomplete melting of these particles.

The size of spherical Type-B particles (> 50 μm) was considerably larger than that of spherical Type-A particles (~ 1 to 10 μm), which should be attributed to the formation process. Type-A particles were formed by the condensation of SiO vapor^9^. On the contrary, the large size of spherical Type-B particles could not be explained by a similar condensation process from gaseous phase. We speculated that the spherical Type-B particles were formed by the cooling of the melted droplet in the free space. High amounts of Ca, Al, and titanium (Fig. 3), which are refractory elements remaining in slag particles in coal and steel plants, in Type-B particles supported their formation from melt. Moreover, spherical slag was formed by their solidification in the free space^28,29^. Thus, the formation process could explain the concentration and morphology of Type-A and -B particles.

This study provided the chemical and morphological characteristics of 67 CsMPs, the number of which was larger than that in previous CsMP studies (less than 10 particles). In addition, systematic variations found in the relationships of ^137^Cs radioactivity with (i) the volume of CsMPs (Fig. 5a), (ii) porosity (Fig. 5b), (iii) Rb/Sr ratio (Fig. 6a), and (iv) ^125^Sb activity (Fig. 6b) were shown. They contributed to the elucidation of additional information on the formation and emission processes of Type-A and -B particles from Units 2/3 and 1, respectively. The 3D structure obtained via µ-X-ray CT showed the difference between Type-A and -B particles, as written below:

(i) Type-B particles had a number of voids, and their ^137^Cs concentration was 10,000 times lower than that of Type-A particles because of their difference in generation processes. That is, condensation of gaseous species and melt solidification were the main processes for Type-A and -B particles, respectively.
(ii) Among Type-B particles, spherical particles had higher ^137^Cs and ^125^Sb concentrations and Rb/Sr ratio than non-spherical particles. This difference was possibly due to the fast cooling process, which inhibited the loss of volatile species.
(iii) These findings suggested that Type-A and -B particles differed in terms of chemical and physical properties due to their diverse formation processes. They contributed to the enhanced understanding of the inner condition of the units of FDNPP and their behavior in the environment.

## Methods

### Sample preparation

Road dust samples were collected at more than 100 sites within 45 km around FDNPP during 2011–2012^18^. These samples had high radiation doses of ~ 20 to 100 μSv/h. Details regarding these materials have been described by Yamamoto et al.^18^ Non-woven fabric cloths used as ground cover materials for vegetable cultivation at 50 km west from FDNPP were also used, the details of which should be referred to Yamaguchi et al.^8^. Fifty-seven Type-B particles were collected from the road dust samples, and 10 Type-A particles were collected from the road dust samples and non-woven fabric cloths. A list of separated Type-A and -B particles is shown in Table S1 in Supporting Information.

The spatial distribution of radioactive Cs in the road dust samples and non-woven fabric cloths was measured through autoradiography with imaging plates (IP; BAS-MS 2040, Fujifilm Corp., Japan) and an IP reader (FLA-9000, Fujifilm Corp., Japan). High-radioactivity spots were identified in the image obtained using the IP reader (Supplementary Fig. S4). These spots were considered CsMPs. The wet separation method^16,17^ enabled us to separate CsMPs from the samples (Supporting Information). After the separation, the CsMPs in the water used for the separation were carefully dropped on Kapton tape and air-dried for SEM (S-4500, Hitachi, Japan) with EDS (Sigma, Kevex, USA) measurement to observe their shape and elemental composition.

### Measurements of ^134^Cs, ^137^Cs, and ^125^Sb activities

The ^134^Cs (604.7 keV) and ^137^Cs (661.7 keV) activities in the identified CsMPs were determined by gamma-ray spectrometry with a HPGe (GX4018, CANBERRA Industries Inc., USA) for each CsMP to determine the unit of FDNPP from which the CsMPs originated.

Radioactivity standard solutions for ^134^Cs (0.182 Bq as of November 25, 2016, Japan Radioisotope Association, CZ-010) and ^137^Cs (1.40 Bq as of Novemver 25, 2016, Japan Radioisotope Association, CS-005) dispersed on a filter with 1 mm square were used for the calibration of the gamma-ray spectrometer. These radioactivity standard solutions were calibrated using the Japan Calibration Service System (JCSS; https://www.nite.go.jp/en/iajapan/jcss/index.html). The activity of ^125^Sb (*T*_1/2_ = 2.7 y) in the particles was determined using 600.6 keV gamma-ray from ^125^Sb. The detection efficiency of the gamma-ray of the present detector was estimated using the efficiency curve constructed using the relative counting efficiencies for plural gamma-rays from europium-152 (^152^Eu) and the normalizing point at 661.7 keV gamma-ray from ^137^Cs. The relative counting efficiencies were obtained with the ^152^Eu source, which was placed in a small plastic tablet and is commercially available as an energy calibration source. The detection efficiency of the gamma-ray from the ^137^Cs obtained via the abovementioned method was used as the normalizing point. The logarithmic relationship between the relative counting efficiency and the gamma-ray energy was established to fit the power function by using the least squares method and normalized at 661.7 keV.

### Micro-X-ray CT analysis

For Type-B particles with large particle sizes, µ-X-ray CT analysis was conducted at BL37XU of SPring-8 (Hyogo, Japan). White X-ray was monochromatized using a double-crystal monochromator with a Si(111) plane. The range of X-ray energy was 6–37.7 keV, and Rh coating flat mirrors were used to eliminate high-order harmonics. In this study, 7.100 (below Fe, K-edge emission), 7.131 (above Fe K-edge emission), 9.600 (below Zn, K-edge emission), and 9.800 keV (above Zn K-edge emission) were used to obtain 3D images of Fe and Zn. Transmission X-ray images were obtained with a complementary metal–oxide–semiconductor camera with 2048 × 2048 pixels. The number of projections was 3,600 for a 360° rotation with 30 dark images and 30 images without sample. The measurement time was approximately 1 min per particle with on-the-fly rotation. The effective pixel size (spatial resolution) was 0.65 μm^30^. For this measurement, Kapton tape with Type-B particles was cut into small sizes (~ 0.5 × 0.5 mm^2^) and attached on the polyimide tube that was established vertically on the metallic base. Reconstruction was performed using the convolution back-projection method^31^. After reconstruction, the images were converted into binary images on the basis of a threshold for the intensity of each pixel to distinguish the particles from the background (= void space) via Image J^32^ for calculating the particle volume (Supplementary Fig. S5). The binary image enabled us to calculate the area of particles for each slice. The spatial resolution of the image of a slice was 0.65 × 0.65 μm^2^, and the distance among slices was 0.65 μm. Thus,

$$V ({\mathrm{m}\mathrm{m}}^{3}) = P \times {\left(0.65\right)}^{3},$$

where *V* and *P* are the volume of the sample and the total pixels of the sample area in all slices, respectively. In this calculation, the voids of Type-B particles were excluded from the volume. The porosity of Type-B particles was also calculated using binary images. We could fill the holes of the binary images by using Image J to obtain the whole particle volume and calculate porosity (Supplementary Fig. S5). The detection limit size of the voids was 0.65 µm in diameter.

We could acquire 3D projection, including the information of the inner structure. An elemental map could also be generated by observing the difference between the pre- and post-edge energies of an element. In this study, projections below and above Fe and Zn K-edges were recorded to obtain Fe and Zn mapping.

### Micro-XRF analysis

For Type-B particles, μ-XRF measurement for various elements was conducted at BL-4A of the Photon Factory PF, KEK (Tsukuba, Japan). White X-ray was monochromatized using a Si double-crystal monochromator with a Si (111) plane and focused to ~ 5 × 5 μm^2^ at BL-4A^33^. Fluorescence yield mode was adopted for the samples. Fluorescent X-ray was detected using a four-element Si drift detector. The samples were placed at 45° from the incident beam. XRF showed the 2D elemental mapping with an X–Y axis stepping-motor-driven stage. Incident X-ray at 16.2 keV was used to include Sr Kα emission for XRF measurement.

## Supplementary information

Supplementary information 1.

Supplementary information 2.

Supplementary information 3.
