# Supplementary material for: Characterization of two types of cesium-bearing microparticles emitted from the Fukushima accident via multiple synchrotron radiation analyses
Source: Sci Rep. 2020 Jul 21;10:11421. doi: 10.1038/s41598-020-68318-2 (PMC7374699; doi:10.1038/s41598-020-68318-2)
Supplement: Supplementary file 3 — Supplementary information 3. [file 41598_2020_68318_MOESM3_ESM.pdf]

Supporting Information

For

Characterization of two types of cesium-bearing microparticles emitted from the Fukushima accident via multiple synchrotron radiation analyses

by

Hikaru Miura, Yuichi Kurihara, Masayoshi Yamamoto,
Aya Sakaguchi, Noriko Yamaguchi, Oki Sekizawa, Kiyofumi Nitta,
Shogo Higaki, Daisuke Tsumune, Takaaki Itai, and Yoshio Takahashi

Details of the wet separation method to separate the CsMP

A sample (soil, filter, cloth sand, and so on) including the CsMP was cut and placed into a plastic tube with water. Subsequently, ultrasonic agitation was performed to separate CsMP from the sample. At this point, the CsMP was transferred to the water phase as suspended particulate matter along with many other particles. The water containing the CsMP was divided into two tubes; one of the two exhibited higher ^{137}Cs radioactivity due to the presence of CsMP, which can be identified with the NaI scintillation counter (Packard Cobra 5003, GMI, USA) in a relatively short time compared with the detection using the HPGe. This separation process using water was adopted assuming that CsMPs are water-insoluble. The separation process was repeated approximately 30 times. After the final separation, the CsMP with water was loaded on carbon tape and air-dried. More details regarding this wet separation method are given in Kurihara et al.[17] This method can separate CsMPs more quickly and efficiently than previous methods.[4,8] In addition, identifying CsMPs separated by this method using SEM is easier because the method does not use any adhesive tapes that can incorporate CsMPs.

Table S1 List of CsMPs.[4,6,9,22]

Name	$^{134}Cs/^{137}Cs$	^{137}Cs / Bq	^{125}Sb / Bq	Rb/Sr	Pb/Sr	Shape	Size / mm^3	Porocity / %
F4-F5-1	0.950 ± 0.054	3.63 ± 0.03	N.D.	0.086	0.57	Non-spherical	3.3.E-04	24
F4-F5-2	0.967 ± 0.052	4.60 ± 0.03	N.D.	-	-	Non-spherical	-	-
F4-F5-3	0.962 ± 0.039	13.3 ± 0.08	N.D.	0.091	3.0	Non-spherical	5.3.E-04	17
F4-F5-4	0.967 ± 0.045	31.6 ± 0.20	N.D.	-	-	App-spherical	1.3.E-03	19
F4-F5-5	0.912 ± 0.049	3.23 ± 0.02	N.D.	0.10	3.3	Non-spherical	3.4.E-05	17
F6-F10-1	0.944 ± 0.042	18.4 ± 0.1	N.D.	0.40	19	Spherical	8.3.E-05	42
F13-F30-1	0.948 ± 0.034	81 ± 0.4	N.D.	-	-	Non-spherical	2.1.E-03	15
F13-F30-2	0.970 ± 0.026	53.1 ± 0.21	N.D.	0.067	0.64	Non-spherical	4.9.E-03	11
F13-F30-3	0.968 ± 0.020	251 ± 0.78	N.D.	0.089	0.63	Non-spherical	4.2.E-03	15
F13-F30-4	0.964 ± 0.041	116 ± 0.75	N.D.	0.061	0.31	Non-spherical	2.4.E-02	21
F13-F30-5	0.969 ± 0.032	37.0 ± 0.17	N.D.	0.11	0.89	Non-spherical	9.6.E-04	22
F13-F30-6	0.944 ± 0.027	144.79 ± 0.60	N.D.	-	-	Non-spherical	-	-
F13-F30-7	0.952 ± 0.043	6.28 ± 0.04	N.D.	0.088	0.56	Non-spherical	5.5.E-04	11
F13-F30-8	0.947 ± 0.029	114 ± 0.53	N.D.	0.30	2.6	Non-spherical	2.3.E-03	4.6
F13-F30-9	0.960 ± 0.043	6.58 ± 0.04	N.D.	0.072	0.35	Non-spherical	9.2.E-04	6.4
F13-F30-10	0.966 ± 0.021	214 ± 0.72	N.D.	0.21	2.1	Non-spherical	3.8.E-03	7.7
F13-F30-11	0.969 ± 0.027	10.71 ± 0.04	N.D.	-	-	Non-spherical	-	-
F13-F30-12	0.976 ± 0.020	264.66 ± 0.81	0.8 ± 0.1	-	-	Non-spherical	-	-
F13-F30-13	0.974 ± 0.032	35.7 ± 0.17	N.D.	0.076	0.34	Non-spherical	1.9.E-03	11
F13-F30-14	0.963 ± 0.013	183.77 ± 0.34	1.2 ± 0.1	-	-	Non-spherical	-	-
F13-F30-15	0.952 ± 0.014	166.65 ± 0.32	1.0 ± 0.1	-	-	Non-spherical	-	-
F13-F30-16	0.967 ± 0.015	139.77 ± 0.29	1.0 ± 0.1	-	-	Non-spherical	-	-
F13-F30-17	0.963 ± 0.005	13199 ± 9	97.2 ± 2.1	0.69	5.0	Spherical	6.2.E-03	37
F13-F30-18	0.946 ± 0.019	85.75 ± 0.23	N.D.	-	-	Non-spherical	-	-
F13-F30-19	0.967 ± 0.014	120.32 ± 0.24	N.D.	-	-	Non-spherical	-	-
F13-F30-20	0.961 ± 0.014	135.73 ± 0.26	N.D.	-	-	Non-spherical	-	-
F13-F30-21	0.950 ± 0.014	121.28 ± 0.24	N.D.	-	-	Non-spherical	-	-
F13-F30-22	0.986 ± 0.044	94.7 ± 0.49	N.D.	0.074	0.34	Non-spherical	7.6.E-03	18
F13-F30-23	0.963 ± 0.008	2257 ± 0.5	N.D.	0.73	33	Spherical	1.4.E-02	53
F13-F30-24	0.962 ± 0.015	108 ± 0.17	0.5 ± 0.0	-	-	Non-spherical	-	-
F13-F30-25	0.967 ± 0.008	1412 ± 1	4.8 ± 0.2	0.13	5.0	App-spherical	1.8.E-02	41
F13-F30-26	0.963 ± 0.007	2036 ± 1.60	7.1 ± 0.2	0.14	2.5	Non-spherical	3.0.E-02	29
F13-F30-27	0.963 ± 0.004	1476 ± 0.61	5.1 ± 0.1	0.19	1.8	Non-spherical	4.0.E-02	17
F13-F30-28	0.964 ± 0.005	1033 ± 0.51	3.3 ± 0.1	0.072	0.45	Non-spherical	-	-
F13-F30-29	0.962 ± 0.005	1042 ± 0.51	2.3 ± 0.1	-	-	Non-spherical	-	-
F13-F30-30	0.968 ± 0.008	909 ± 0.81	4.3 ± 0.1	0.10	3.3	Non-spherical	1.1.E-02	3.7
F13-F30-31	0.965 ± 0.005	10567 ± 6	26.4 ± 0.8	0.38	5.1	App-spherical	2.5.E-02	52
F13-F30-32	0.966 ± 0.007	2508 ± 0.84	9.7 ± 0.2	0.066	0.49	Non-spherical	9.0.E-02	23
F13-F30-33	0.963 ± 0.008	2185 ± 1.91	6.5 ± 0.3	0.17	5.8	Non-spherical	2.3.E-02	22
F13-F30-34	0.957 ± 0.008	752 ± 1	2.7 ± 0.1	-	-	Non-spherical	1.6.E-02	3.7
F13-F30-35	0.968 ± 0.008	1125 ± 1.06	5.7 ± 0.2	-	-	Non-spherical	-	-
F13-F30-36	0.966 ± 0.003	3316 ± 0.92	10.1 ± 0.2	-	-	Non-spherical	-	-
F13-F30-37	0.961 ± 0.004	1961 ± 0.79	6.3 ± 0.1	0.068	0.86	Non-spherical	6.7.E-02	34
F13-F30-38	0.963 ± 0.005	1115 ± 0.53	2.4 ± 0.1	1.0	31	Spherical	1.4.E-02	10
F13-F30-39	0.970 ± 0.008	1156 ± 1.1	3.4 ± 0.1	0.22	6.7	Spherical	1.2.E-02	39
F13-F30-40	0.964 ± 0.004	1653 ± 0.65	8.0 ± 0.1	0.079	1.9	Non-spherical	6.9.E-02	21
F14-F22-1	0.962 ± 0.022	197 ± 1.00	N.D.	-	-	Spherical	1.5.E-03	8.5
F15-F24-1	0.966 ± 0.047	52.4 ± 0.4	N.D.	-	-	Non-spherical	1.1.E-04	7.6
F16-F26-1	0.947 ± 0.046	46.5 ± 0.3	N.D.	0.13	2.7	Spherical	5.5.E-04	32
F17-F28-1	0.949 ± 0.030	4.42 ± 0.02	N.D.	-	-	Spherical	8.8.E-05	11
N6-N33-1	0.952 ± 0.030	8.57 ± 0.03	N.D.	0.43	5.8	Spherical	3.6.E-05	11
N7-N35-1	0.983 ± 0.033	8.68 ± 0.04	N.D.	0.14	3.0	Spherical	2.3.E-04	23
S2-K7-1	0.938 ± 0.031	33.3 ± 0.2	N.D.	0.61	21	Spherical	1.3.E-04	2.9
S2-K7-2	0.944 ± 0.031	11.8 ± 0.1	N.D.	-	-	Spherical	2.6.E-04	18
S3-R7-1	0.939 ± 0.045	10.9 ± 0.07	N.D.	0.14	2.7	Spherical	2.3.E-04	35
S3-R7-2	0.950 ± 0.050	7.99 ± 0.06	N.D.	-	-	Spherical	-	-
S9-R3-1	0.943 ± 0.030	4.37 ± 0.02	N.D.	0.65	35	Spherical	1.3.E-05	0.78
N11-N26-1	1.078 ± 0.013	62 ± 0.12	-	-	-	Non-spherical	3.3.E-08	-
O8-O23-1	1.092 ± 0.049	1.47 ± 0.01	-	-	-	Spherical	4.0.E-09	-
O9-O26-1	1.082 ± 0.064	2.01 ± 0.02	-	-	-	Spherical	6.0.E-09	-
O10-O31-1	1.093 ± 0.022	21.70 ± 0.07	-	-	-	Spherical	4.8.E-08	-
S11-R6-1	1.089 ± 0.042	2.32 ± 0.01	-	-	-	Spherical	7.0.E-09	-
T15-1	1.046 ± 0.062	1.36 ± 0.01	-	-	-	Spherical	5.0.E-09	-
T15-2	1.041 ± 0.059	0.72 ± 0.01	-	-	-	Spherical	1.0.E-09	-
nwc-1	1.070 ± 0.077	0.89 ± 0.01	-	-	-	Spherical	1.0.E-09	-
nwc-2	1.097 ± 0.071	0.68 ± 0.01	-	-	-	Spherical	2.0.E-09	-
nwc-3	1.081 ± 0.119	0.67 ± 0.01	-	-	-	Spherical	4.0.E-09	-
Particle 1	-	3.31 ± 0.06	-	-	-	Spherical	9.2.E-09	-
Particle A	-	1.29 ± 0.02	-	-	-	Spherical	4.2.E-09	-
Particle B	-	1.49 ± 0.03	-	-	-	Spherical	1.1.E-08	-
Particle C	-	1.10 ± 0.02	-	-	-	Spherical	1.4.E-09	-
Particle A	-	34.79 ± -	-	-	-	Spherical	4.8.E-08	-
Particle B	-	157.66 ± -	-	-	-	Spherical	1.4.E-07	-
Particle C	-	20.99 ± -	-	-	-	Spherical	3.4.E-08	-
Particle D	-	6.11 ± -	-	-	-	Spherical	1.4.E-08	-
KOI	-	11.30 ± 0.15	-	-	-	Spherical	2.1.E-08	-
OTZ	-	2.07 ± 0.031	-	-	-	Spherical	4.2.E-09	-
AQC	-	0.91 ± 0.04	-	-	-	Spherical	1.9.E-08	-
OTZ10	-	1.94 ± 0.031	-	-	-	Spherical	5.6.E-09	-

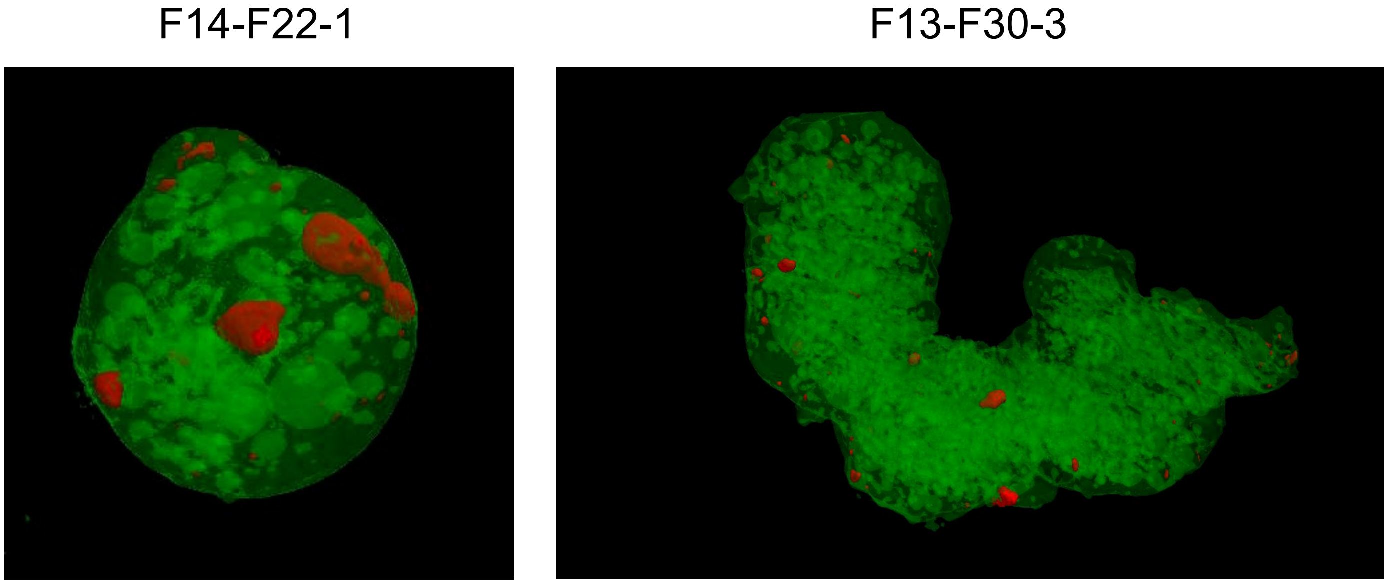

Figure S1
3D translucent μ-X-ray CT image for spherical (left) and non-spherical (right) Type-B particles. The red areas indicate Fe-rich parts.

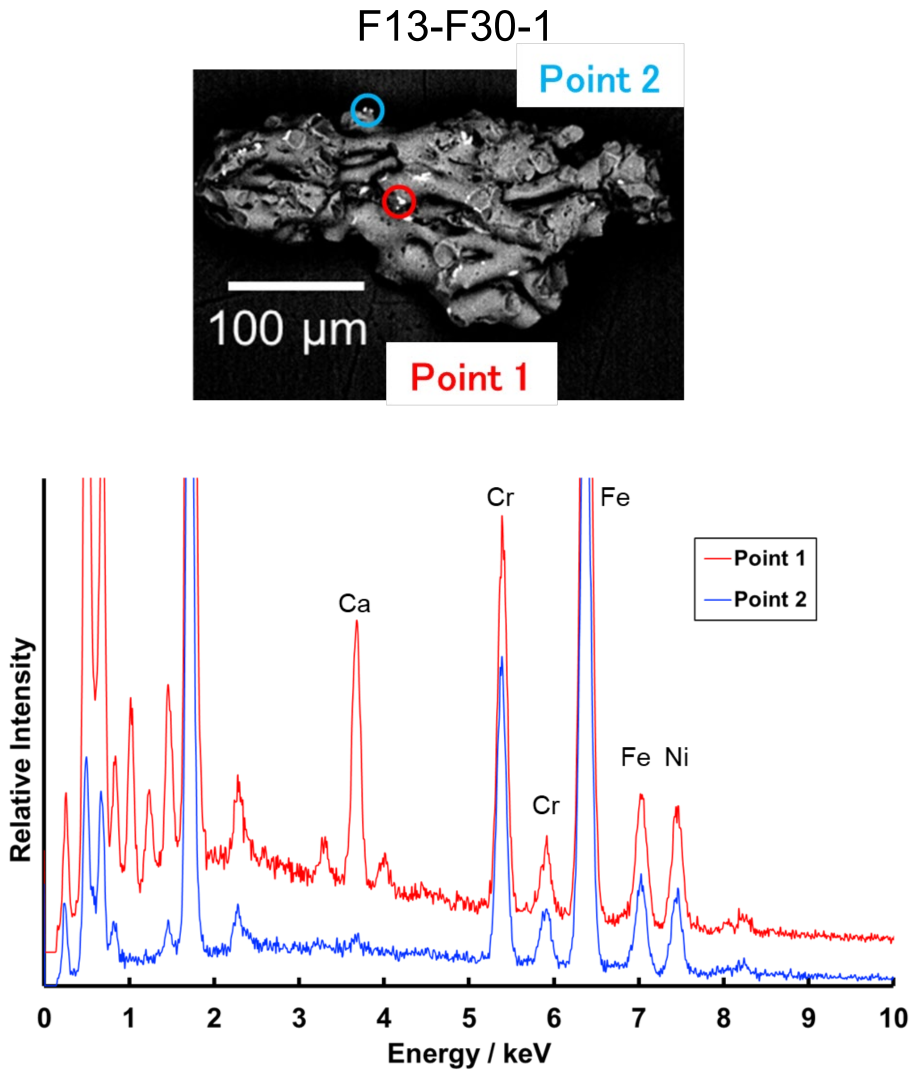

Figure S2
The EDS results of the white area of the Type-B particle identified by backscattered electron image. We can observe Cr and Ni peaks in the Fe-rich part, which is suggested to be originated from stainless steel in Unit 1.

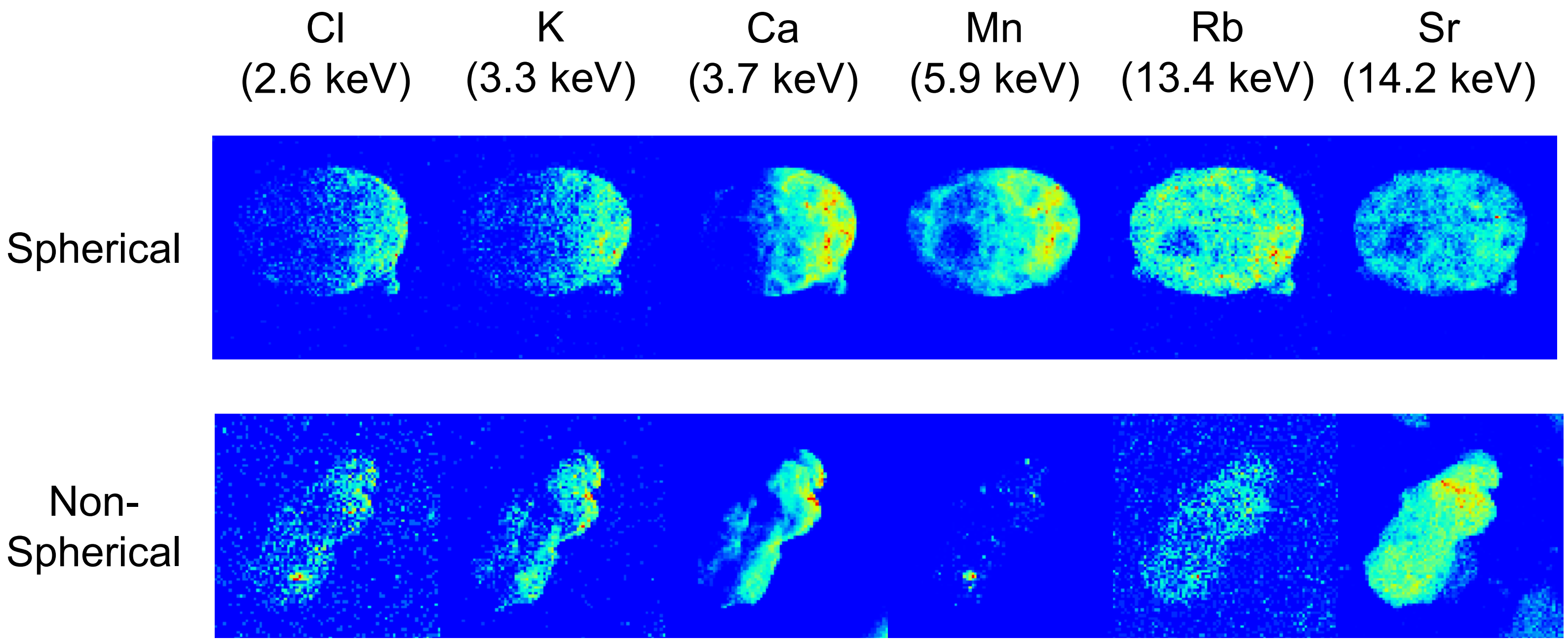

Figure S3
XRF elemental 2D maps of spherical and non-spherical Type-B particles.

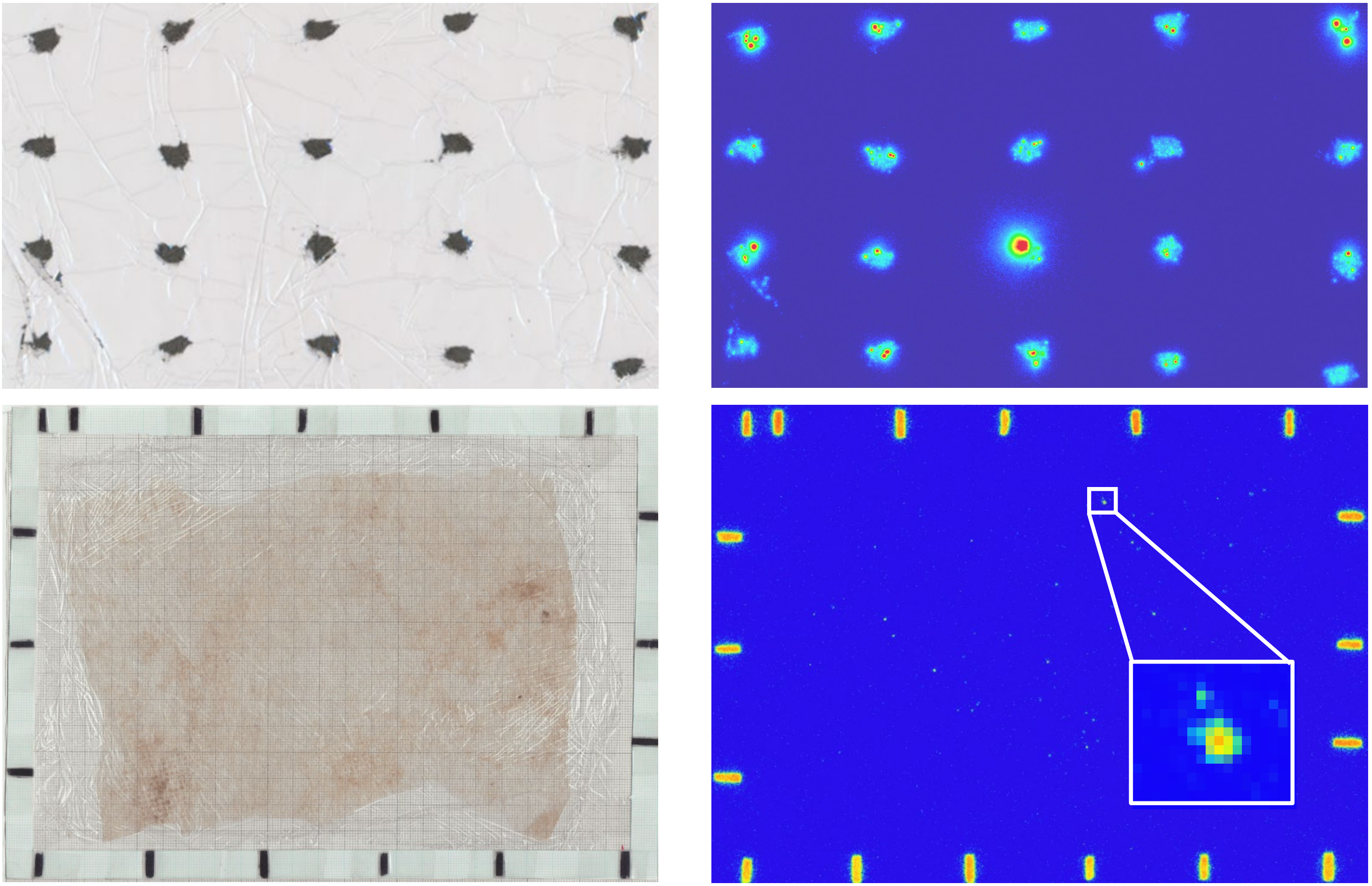

Figure S4

Left figures are road dusts (~100 mg × 20; upper left) and non-woven fabric cloth (lower left) loaded on IP. Right figures are autoradiography images of the dusts (upper right) and cloth (lower right). High radioactivity spots are considered to be Type-A or Type-B particles.

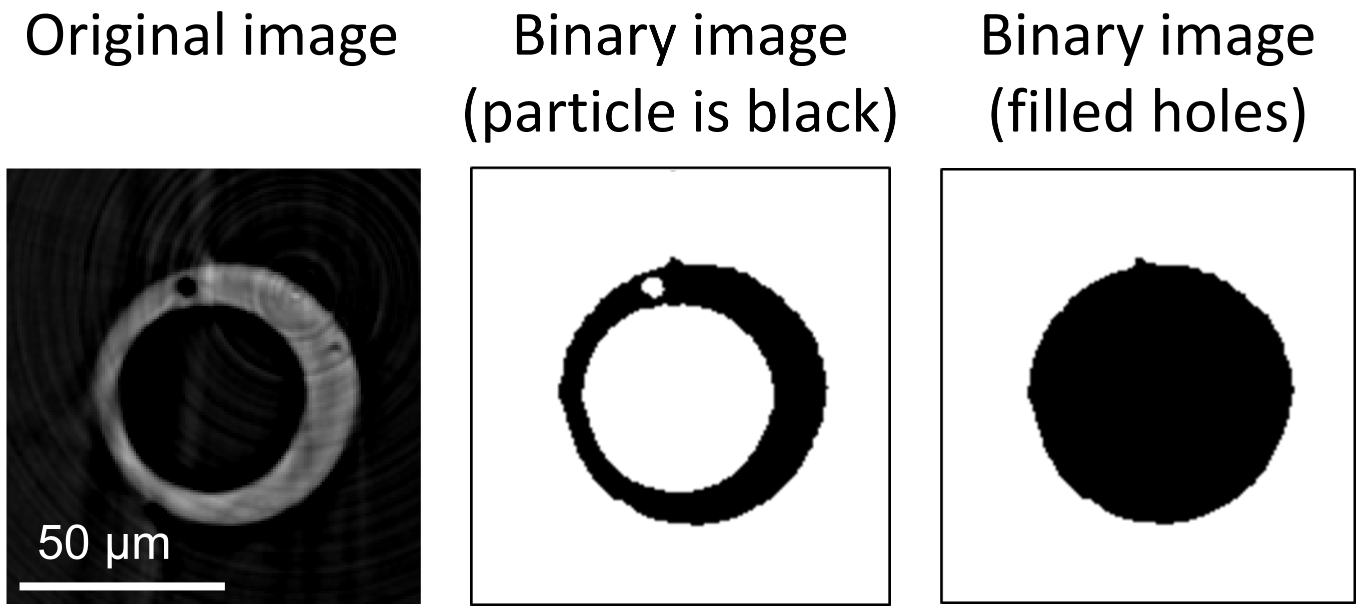

Figure S5
Left image is an original slice of reconstructed µ-X-ray CT image for spherical Type-B particles. Center image is a binary image converted from the left image using a threshold to distinguish the particle and background. Right image is a binary image with filled holes. Porosity was calculated by the center and right images.
